# Violent Behavior Is Associated With Emotion Salience Network Dysconnectivity in Schizophrenia

**DOI:** 10.3389/fpsyt.2020.00143

**Published:** 2020-02-28

**Authors:** Andràs Tikàsz, Stéphane Potvin, Jules R. Dugré, Cherine Fahim, Vessela Zaharieva, Olivier Lipp, Adrianna Mendrek, Alexandre Dumais

**Affiliations:** ^1^Centre de recherche de l'Institut Universitaire en Santé Mentale de Montréal, Montreal, QC, Canada; ^2^Department of Psychiatry, University of Montreal, Montreal, QC, Canada; ^3^Centre de recherche du CHU Ste-Justine, Montreal, QC, Canada; ^4^Montreal Neurological Institute and Hospital, McGill University, Montreal, QC, Canada; ^5^Department of Psychology, Bishop's University, Sherbrooke, QC, Canada; ^6^Institut Philippe-Pinel de Montréal, Montreal, QC, Canada

**Keywords:** schizophrenia, violence, negative emotions, anterior cingulate cortex, functional connectivity, fMRI

## Abstract

**Background:** Despite individuals with schizophrenia being at an elevated risk of violence compared to the general population, limited efforts have been invested in investigating the neurobiological etiology explaining the increase. Among the few studies examining functional disruptions pertaining to violent schizophrenia patients using fMRI, only one study has considered functional connectivity. The current state of knowledge does not allow to infer deficits in functional connectivity specific to distinct cognitive/emotional states that have been associated with the emergence of violence in schizophrenia, such as negative emotion processing. This study sought to identify disrupted connectivity among men with schizophrenia and a history of violence (SCZ+V), compared to men with schizophrenia without a history of violence (SCZ-V) and healthy controls, during negative emotion processing using fMRI.

**Methods:** Twenty SCZ+V, 19 SCZ-V, and 21 healthy men were scanned while viewing negative images.

**Results:** Negative images elicited an increased connectivity between the dorsal anterior cingulate cortex (dACC) and the bilateral rostral prefrontal cortex (rPFC), as well as a decreased functional connectivity between the frontal regions (bilateral rPFC and dACC) and the putamen and hippocampus in SCZ+V men as compared to SCZ-V men and healthy controls. Concurrently, the centrality of the dACC within the network was reduced in SCV+V subjects.

**Conclusions:** These results suggest an inefficient integration of the information by the dACC between frontal and limbic regions in SCZ+V men during negative emotion processing and highlight the importance of the ACC in the neurobiological bases of violent behavior in schizophrenia.

## Introduction

Although individuals with schizophrenia (SCZ) are at an increased risk of presenting violent behaviors compared to the general population (1, 2), the current understanding of the neurobiological mechanisms underlying the emergence of such behaviors is limited (3). A defective processing of negative emotions such as fear and anger in violent individuals has been proposed (4–6), as these negative emotions were shown to precipitate violence and aggression (7, 8). Considering that the system of structures involved in the experience, processing, and regulation of emotions include the prefrontal cortex, amygdala, hippocampus, insula, striatum and anterior cingulate cortex (ACC), Davidson et al. (9) have suggested that dysfunctions within this system might be implicated in the failure to regulate negative emotions and potentially contribute to an increased predisposition for aggressive and violent behaviors. Given the growing association between negative affect and violent behavior in the context of psychotic disorders (8, 10, 11), similar abnormalities might be expected in violent men with (SCZ+V) or without schizophrenia (12–14).

Structural alterations within the emotional-salience network of violent SCZ patients have generally supported this assumption (15–17). Reduced brain volumes in the hippocampus (18–20) parahippocampus (20), amygdala (21), ACC (22), orbitofrontal cortex (19), and cerebellum (23), and increased gray matter volumes in the putamen (18) have been observed among SCZ+V subjects. Increased gray matter volumes in the hypothalamus and right precuneus in SCZ men with a history of conduct disorder (24), and larger orbitofrontal cortex (25) and caudate volumes (26) amongst SCZ men with high levels of aggressive behavior have also been reported. However, these structural differences do not provide an insight into the alterations involved in the processing of stimuli that may trigger violent behavior.

Few functional neuroimaging studies have examined emotion processing among men with SCZ in relation to violence/aggression or traits related to them (17, 27). Dolan and Fullam (28) observed blunted amygdala response to fearful faces in SCZ men with high levels of psychopathic traits in comparison to SCZ men with low psychopathic traits. Kumari et al. (5) reported greater activity in the thalamus and the caudate nucleus in response to threat stimuli in SCZ+V men relative to non-psychotic individuals diagnosed with antisocial personality disorder. Recent work by our laboratory have found a hyperactivated ventral ACC during the processing of negative emotion (29), and a deficit in dorsolateral prefrontal cortex activation when inhibiting a response while viewing angry faces (30) in schizophrenia individuals with a history of violent/aggressive behavior relative to both non-violent SCZ (SCZ-V) and healthy subjects. These results suggest abnormalities in the emotional-salience network that encompass regions involved in emotion regulation (9) among men with SCZ+V during the processing of negative emotions.

Hoptman et al. (31) have authored the sole study to date to investigate functional connectivity in relation with violent/aggression among SCZ subjects. They reported reduced functional connectivity at rest between the amygdala and ventral prefrontal regions in male schizophrenia patients, which correlated inversely with aggression. These results, although concordant with current conceptualizations of the neurobiological basis of violence implying a disrupted fronto-limbic network (6, 14, 15, 27, 32), do not allow to infer deficits specific to distinct cognitive/emotional states that have been associated with the emergence of violence in SCZ, such as negative emotion processing and regulation (4, 8, 9). Moreover, their results were based on the analysis of single pairs of regions, although the neurobiology of violence is likely to involve a larger set of brain regions from the emotion salience network. Nevertheless, the seminal work of Hoptman et al. (31), as well as previous work in functional and structural imaging in SCZ+V, are an incentive to investigate broader functional connectivity disruptions within the emotional-salience network during the processing of negative emotions in a population of SCZ with a reported history of serious violence.

Currently, the understanding of the neural underpinning of emotion processing in SCZ+V is limited. Therefore, using adequate control groups, this study sought to identify disrupted functional connectivity within the emotional-salience network among men with SCZ+V, compared to men with SCZ-V and healthy controls, during negative emotion processing. We hypothesized that fronto-limbic functional connectivity will be reduced in SCZ+V men compared to the two other groups.

## Methods

### Participants

A total of 60 male participants were recruited for this study. Based on the *Diagnostic and Statistical Manual of Mental Disorders IV* (DSM-IV) criteria, thirty-nine outpatients with schizophrenia (SCZ) or schizo-affective disorder were included. Nineteen patients without (SCZ-V) and 20 patients with (SCZ+V) a history of serious violent behavior were recruited from general and forensic psychiatric hospitals, respectively. Serious violence was defined as “a history of armed aggression resulting in injuries or death” (29) and was assessed based on the *Structured Clinical Interview for DSM-IV* (SCID-IV) (33), the *MacArthur Community Violence Instrument* (34), and clinical files. The number of DSM-IV diagnostic criteria for antisocial personality disorder met by participants with a history of violence were calculated. All patients were stabilized on antipsychotic medication (no changes for >2 months) prior to participating in the study. Antipsychotic dosage was compared between patients using chlorpromazine equivalents (35). Symptom severity was evaluated with the *Positive And Negative Syndrome Scale* (36) yielding five sub-scores: positive, negative, disorganization/cognitive, excitation, and depression (37). Urine drug screenings were administered. Twenty-one healthy men with no history of violent behavior or mental disorders were recruited. Healthy participants were screened with the non-patient edition of the SCID-IV (33).

Participants were free of concomitant neurological disorders, substance use disorders (lifetime for Healthy subjects; last 12 months for SCZ), or magnetic resonance imaging (MRI) contra-indications. Participants had an IQ > 70 (38). Parental socio-economic status (SES) was assessed according to the *National Occupational Classification* (39). Informed consent was obtained from all individual participants included in the study.

All procedures performed in studies involving human participants were in accordance with the ethical standards of the institutional research committee from the *Regroupement de Neuroimagerie du Québec*, the *Centre de recherche de l'Institut Universitaire en Santé Mentale de Montréal*, and the *Institut Philippe-Pinel de Montréal*. All subjects gave written informed consent in accordance with the 1964 Declaration of Helsinki.

### Experimental Procedure and Task

While in the scanner, participants were shown blocks of emotional pictures taken from the *International Affective Picture System* (IAPS) (40). Based on IAPS normative data, the pictures were grouped by valence (i.e., negative, positive, neutral) and arousal intensity (i.e., high arousal, low arousal), yielding 5 conditions: (i) high arousal positive, (ii) high arousal negative, (iii) low arousal positive, (iv) low arousal negative, and (v) neutral. Each condition was presented in two separate 48.5 s blocks, except for the neutral block which was presented four times. Each block contained 10 images from the same condition, each image appearing for 3 s followed by a fixation point presented on a blank screen for 1.75 s (ITI: 4.75 s). Blocks were interceded by 16 s rest periods, and the order of the blocks was pseudo-randomized. Preceding each block was a 3 s period when participants were instructed on screen to press a button whenever they saw a person in the picture. This measure was to ensure participants were attentive to the content of the images they were presented. The task was explained in detail to the participants prior to undergoing the experiment, and they received no additional training outside of the scanner.

Following the fMRI session, participants rated the images from 0 to 8, corresponding to an absence of any emotional reaction to the strongest emotion ever felt, respectively.

### MRI Data Acquisition Parameters

All images were acquired on a 3.0 Tesla TRIO-TIM system. Blood oxygen level dependent (BOLD) signal was recorded using a T2-weighted gradient echo-planar imaging (EPI) sequence with an inline retrospective motion correction algorithm. Forty-one axial slices angled parallel to the AC-PC line were acquired (TR: 3,000 ms), with 3.5 mm isotropic voxels, yielding a 64 × 46 matrix. Using a 3D spoiled gradient echo sequence, 176 co-planar sagittal anatomical slices with 1 mm isotropic voxels were also acquired, yielding a 256 × 256 matrix.

### fMRI Data Preprocessing

fMRI data was analyzed with the CONN v.17 functional connectivity toolbox (41). The default preprocessing pipeline was utilized, constituted of SPM v.12 functions (42). Functional images were realigned, centered, slice-time corrected, corrected for motion artifacts with the Artifact Detection Toolbox (43), and co-registered to the corresponding anatomical image. The anatomical images were centered, segmented (into gray matter, white matter, and cerebrospinal fluid) and normalized to the *Montreal Neurological Institute* (MNI) stereotaxic space. Functional images were normalized to MNI stereotaxic space using the deformation field from the corresponding anatomical images, spatially smoothed with a 3D isotropic Gaussian kernel (8 mm FWHM), and resliced to 2 mm^3^ voxels.

### Functional Connectivity Analysis

A seed-driven approach was employed for functional connectivity analyses (41). The anatomical component-based noise correction method was used to estimate the physiological BOLD signal noise from the white matter and cerebrospinal fluid (44). These physiological noise processes, together with the 6 realignment parameters and the scans impacted by movement artifacts (scrubbing) were regressed out as first-level nuisance covariates from the BOLD time-series at each voxel. The main activation effects of the conditions were also accounted for to avoid spurious connectivity due to task co-activation (45). Linear detrending was performed. The time-series were band-pass filtered (0.008–0.090Hz), and then weighted by the appropriate HRF-convolved regressor to derive task condition-specific time-series for weighted functional connectivity analyses (41, 46). We limited our primary investigations to the processing of negative pictures (high & low arousal together) based on previous results from our research team (29), which showed the processing of negative emotions to be the most altered in SCZ+V men. Secondary analyses were performed using the positive emotion and neutral conditions.

The 10 seeds/regions of interest (ROIs) were chosen from the Harvard-Oxford atlas. The bilateral rostral prefrontal cortex (rPFC), dorsal ACC (dACC), ventral ACC (vACC), bilateral hippocampus, bilateral putamen, and bilateral amygdala (Table 1) were included based on studies in non-psychotic and SCZ populations that have suggested these regions to be implicated in the neurobiology of violence and aggression (9, 31, 32) including results from our laboratory (30).

**Table 1:** Regions-of-interest used as seed in functional connectivity analysis.

**Seed**	**l/r**	**MNI coordinates**		
		**x**	**y**	**z**
Dorsal Anterior Cingulate Cortex (dACC)	-	0	22	35
Ventral Anterior Cingulate Cortex (vACC)	-	0	21	−15
Amygdala	r	23	−4	−18
Amygdala	l	−23	−5	−18
Hippocampus	r	26	−21	−14
Hippocampus	l	−25	−23	−14
Putamen	r	25	2	0
Putamen	l	−25	0	0
Rostral Prefrontal Cortex (rPFC)	r	32	46	27
Rostral Prefrontal Cortex (rPFC)	l	−32	45	27

*l, left; r, right*.

In the first-level correlation maps, Pearson's correlation coefficients were calculated between the time-course of each pair of ROIs for each subject. Resulting correlation coefficients were converted to normally distributed Z-scores using Fisher's transform to improve second-level General Linear Model analyses (41). Second-level analysis compared the correlation coefficients between groups using the group contrast vector c = [−0.5 −0.5 1] to search for connections that were altered in SCZ+V [1] subjects in comparison to both SCZ-V [−0.5] and Healthy subjects [−0.5].

Second-level analyses were corrected for multiple comparison using a *p*-FDR <0.05 threshold applied at the analysis-level. This is a conservative correction which considers the total number of individual connections in the entire analysis (10 ROIs yielding 45 individual connections) and allows for the identification of between-group differences in the strength of individual connections. To confirm that the effects were specific to SCZ+V subject, *post-hoc* tests were computed for all possible pair-wise comparisons. GIMP was used to build the figures (www.gimp.org).

### Network Analysis

Network analysis was employed to quantitively characterize the between-group differences in topological organization of the functional connectome (47) during the processing of negative emotions. Analyses were done with the GRETNA toolbox (48). The absolute values of the coefficients from the Fisher-Z transformed correlation matrices extracted from CONN were considered, and diagonal entries were set to zero. A cost-threshold range of 0.10-0.34 (i.e., 10% to 34% of the strongest possible connections) with intervals of 0.01 was employed (49–52). Analyses were performed using binary undirected graphs, where connections that met the cost-threshold were set to 1 and all other connections were set to 0. The area under the curve for the range of cost-threshold was calculated for each network metric employed, which provided a cost-integrated value (53) for each subject that was independent of a single threshold selection (49), and could be used for group analyses.

Based on recent studies in schizophrenia (53) and other populations (54), commonly used global metrics were examined, including global and local network efficiency, and regional network metrics, including the betweenness-centrality (reflecting how a brain region is used to enable one area to communicate with another), nodal clustering coefficient (reflecting the level of local connectedness of a node), and nodal local efficiency (a measure of local information transfer). Additional details can be found in Rubinov and Sporns (55).

Cost-integrated metrics were entered in an ANOVA, and *post-hoc* tests were computed for all possible pairwise comparison. Nodes/seeds that showed significant between-group differences in functional connectivity were the focus of the network analysis.

### Clinical Data

Correlation analyses between clinical variables, functional connectivity, and cost-integrated network metrics were calculated. A significance threshold of *p* < 0.05 was implemented.

## Results

### Participant Characteristics

As described in Tikasz et al. (29), no between-group differences were observed for the participants' age, handedness, and ratings of negative images. Furthermore, SCZ+V participants did not differ from SCZ-V participants with regards to diagnosis, age of onset, illness duration, negative symptoms, chlorpromazine equivalents, and treatment with clozapine. However, SCZ+V subjects reported lower parental SES than Healthy subjects, and SCZ+V subjects presented fewer positive and disorganized symptoms than SCZ-V subjects (Table S1). Participants with a history of violence did not report sexual violence. Of the participants reporting violent behaviors, 8 met the criteria for antisocial personality disorder with evidence of conduct disorder in childhood.

### Functional Connectivity Analysis

**Figure 1:**
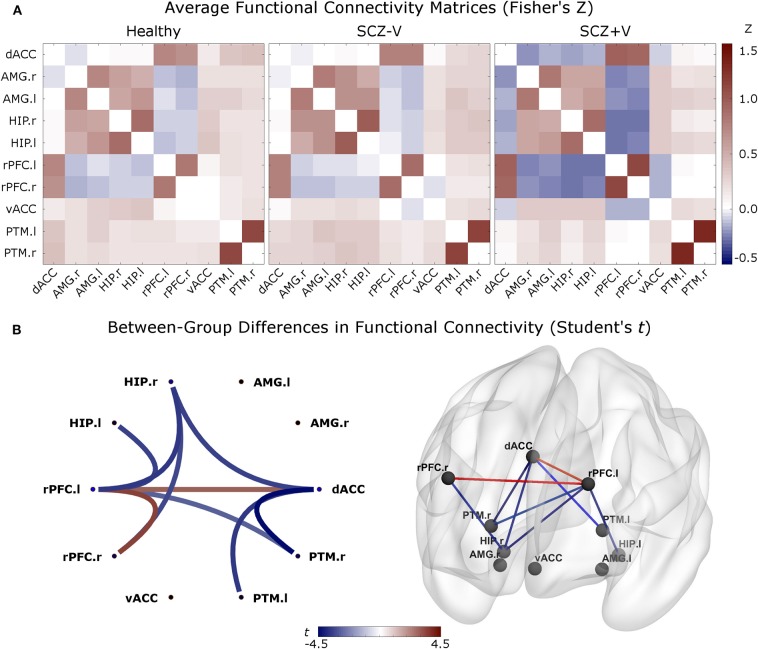
**(A)** Averaged functional connectivity (correlation) matrices (Fisher's Z-score) between each pair of regions for the three groups. The second level analysis was based on these measures. In red: positive correlations; in blue: negative correlations. **(B)** Between-group differences (contrast vector = [−0.5 −0.5 1]) in functional connectivity comparing SCZ+V [1] subjects to SCZ-V [−0.5] and Healthy subjects [−0.5] (*p*-FDR < 0.05 analysis-level). In red: Increased connectivity in SCZ+V subjects, relative to the two other groups; in blue: reduced connectivity in SCZ+V subjects, relative to the two other groups. When positive symptoms were considered as covariates, the reduced connectivity between the left rPFC and the left hippocampus was no longer different between groups. SCZ, schizophrenia; V, violent behavior, dACC, dorsal anterior cingulate cortex; vACC, ventral anterior cingulate cortex; rPFC, rostral prefrontal cortex; PTM, putamen; HIP, hippocampus; AMG, amygdala; r, right; l, left; SCZ, schizophrenia; V, violent behavior.

During negative emotion processing, the SCZ+V group showed distributed alterations in task-modulated functional connectivity (Table 2, Figure 1). In comparison to SCZ-V and Healthy subjects, SCZ+V showed altered patterns of connections at the left and right rPFC, left and right hippocampus, right putamen, and the dACC. Moreover, *post-hoc* analysis revealed that the strength of the individual connections was specifically altered in SCZ+V when compared to *both* SCZ-V and Healthy subjects. The strength of the connectivity between the left rPFC, the right rPFC and the dACC was *increased* in SCZ+V participants compared to both groups. Conversely, in SCZ+V participants, *reduced* connections were observed between: (i) the left rPFC and the bilateral hippocampus, right putamen; (ii) the right rPFC and the right hippocampus; (iii) the dACC and the bilateral putamen, right hippocampus. When positive symptoms were considered as covariates, the reduced connectivity between the left rPFC and the left hippocampus was no longer different between groups. Parental SES, disorganization symptoms, chlorpromazine equivalents and clozapine had no influence on the results (all *p* > 0.05). No between-group differences were observed during positive and neutral emotion processing for any of the connections found to be impaired during negative emotion processing. The group mean Fisher's Z-scores can be found in Table S2.

**Table 2:** Between-group differences [SCZ+V vs. SCZ-V & Healthy] for ROI-to-ROI functional connectivity during negative emotion processing.

**Seed ROI**	**Target ROI**	T^*^	***Post-hoc* LSD**
Putamen r			
	dACC	−4.50	SCZ+V < SCZ-V < Healthy
	rPFC l	−2.92	SCZ+V < SCZ-V & Healthy
dACC			
	Putamen r	−4.50	SCZ+V < SCZ-V < Healthy
	Putamen l	−3.57	SCZ+V < SCZ-V & Healthy
	Hippocampus r	−3.38	SCZ+V < SCZ-V & Healthy
	rPFC l	2.92	SCZ+V > SCZ-V & Healthy
rPFC l			
	Hippocampus r	−3.52	SCZ+V < SCZ-V & Healthy
	rPFC r	3.46	SCZ+V > SCZ-V & Healthy
	Hippocampus l^a^	−3.41	SCZ+V < SCZ-V & Healthy
	dACC	2.92	SCZ+V > SCZ-V & Healthy
	Putamen r	−2.92	SCZ+V < SCZ-V & Healthy
Putamen l			
	dACC	−3.57	SCZ+V < SCZ-V & Healthy
rPFC r			
	rPFC l	3.46	SCZ+V > SCZ-V & Healthy
	Hippocampus r	−3.28	SCZ+V < SCZ-V & Healthy
Hippocampus l			
	rRPFC l^a^	−3.41	SCZ+V < SCZ-V & Healthy
Hippocampus r			
	rPFC l	−3.52	SCZ+V < SCZ-V & Healthy
	dACC	−3.38	SCZ+V < SCZ-V & Healthy
	rPFC r	−3.28	SCZ+V < SCZ-V & Healthy

* *For all between-group differences in this table, a p-FDR <0.05 analysis-level threshold was applied, which takes into account the total number of connections included in this analysis, and allows to identify between-group differences in the strength of individual connections. Post-hoc Least Significant Difference pairwise test confirmed that SCZ+V men were significantly different from both SCZ-V and Healthy men*.

a *When positive symptoms were considered as covariates, the reduced connectivity between the left rPFC and the left hippocampus was no longer different between groups*.

*ACC, Anterior cingulate cortex; FDR, False Discovery Rate; LSD, Least Significant Difference; PFC, prefrontal cortex; ROI, region of interest*.

### Network Analysis

**Figure 2:**
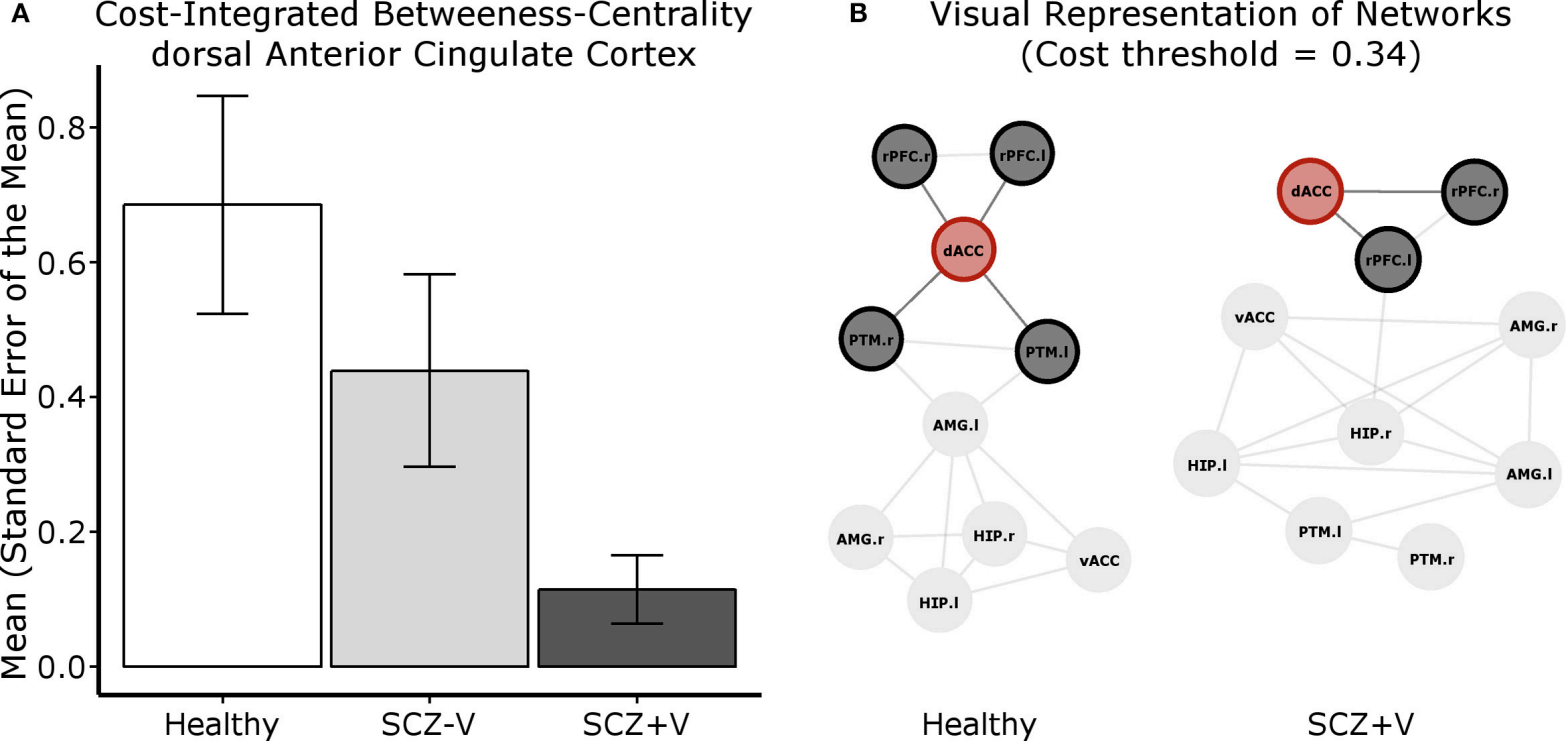
**(A)** Cost integrated betweenness-centrality value for the dACC per group. **(B)** Visual representation of the centrality of the dACC (node in red) within the network of 10 regions investigated. For this representation, Fisher's transformed correlation coefficients were averaged by group, the top 34% coefficients were selected (cost threshold) and binarized. These graphs serve to exemplify that there are more short paths crossing the dACC, therefore holding a higher betweenness-centrality value, in the Healthy subject graph than in the SCZ+V graph. SCZ, schizophrenia; V, violent behavior; dACC, dorsal anterior cingulate cortex; vACC, ventral anterior cingulate cortex; rPFC, rostral prefrontal cortex; PTM, putamen; HIP, hippocampus; AMG, amygdala; r, right; l, left.

The network analysis focused on the seeds with significant between-group differences in connection patterns (Table 2). A significant between-group difference was observed in the betweenness-centrality of the dACC (*F* = 5.04, *p* = 0.010; Figure 2). *Post-hoc* (*p* < 0.05) analysis revealed that the metric was decreased in SCZ+V as compared to Healthy subjects. A linear contrast analysis was subsequently conducted, which revealed the betweenness-centrality of the dACC to decrease linearly with group status (*F* = 10.04, *p* = 0.002), where Healthy>SCZ-V>SCZ+V (Figure 2). Between group-difference for betweenness-centrality was also observed for the left rPFC (*F* = 4.27, *p* = 0.019), where *post-hoc* (*p* < 0.05) analysis indicated that the metric was increased in SCZ+V as compared to SCZ-V and Healthy subjects. Furthermore, significant between-group differences were observed for both the nodal clustering coefficient (*F* = 6.48, *p* = 0.003) and the nodal local efficiency (*F* = 6.29, *p* = 0.003) of the dACC. *Post-hoc* (*p* < 0.05) analysis revealed that these two metrics were increased in SCZ+V in comparison to both Healthy and SCZ-V subjects. No between-group differences were observed for the global network metrics (global and local network efficiency). Parental SES, positive and disorganization symptoms, chlorpromazine equivalents and clozapine had no influence on these results (all *p* > 0.05).

### Clinical Data

In Healthy subjects, a positive correlation was observed between the between-centrality value for the dACC and ratings of negative emotional stimuli (*r* = 0.542; *p* = 0.017), as well as between the nodal local efficiency of the dACC and ratings of emotional stimuli (*r* = 0.532; *p* = 0.019). No correlations were observed between functional connectivity or network metrics and subjective ratings or emotional stimuli and antisocial personality traits in SCZ-V and SCZ+V men.

## Discussion

This is the first fMRI study to investigate task-based functional connectivity during the processing of negative emotions in a sample of men with schizophrenia and a history of violence (SCZ+V), as compared to both non-violent men with (SCZ-V) and without schizophrenia. Viewing negative pictures elicited disrupted functional connectivity in SCZ+V relative to SCZ-V and Healthy subjects in the dACC, bilateral rPFC, right hippocampus and the bilateral putamen. An increased connectivity among SCZ+V men between the dACC and the bilateral rPFC, and concurrently a decreased functional connectivity between the frontal regions (bilateral rPFC and dACC) and the putamen and hippocampus were observed. Furthermore, we observed a gradient effect of group status on the betweenness-centrality of the dACC, as the centrality of the node within the network was reduced in SCZ-V men relative to Healthy subjects, and further reduced in SCZ+V men relative to SCZ-V men. Noteworthy, the connectivity within the emotional salience network during the viewing of positive and neutral pictures did not differ between SCZ+V subjects and the two other groups. Together, these results suggest an inefficient integration of the information by the dACC from frontal and limbic regions in SCZ+V men during negative emotion processing.

The regional betweenness-centrality provides a measure of the capacity of a node to behave as a bridge between the nodes composing the rest of the network, as it is related to the number of shortest paths between two regions passing through. It is a relevant metric within the population investigated, as previous resting-state functional and structural MRI studies have reported a decreased betweenness-centrality (i.e., a less central role within the network) of the ACC in schizophrenia and at-risk populations (53, 56–58). In accord with the extant literature in SCZ subjects, we found a decrease in topological centrality of the dACC in SCZ+V men in comparison to Healthy men. Moreover, we observed a gradient effect between the groups, as the betweenness-centrality of the dACC within the network decreased from Healthy to SCZ-V men, and further decreased from SCZ-V to SCZ+V men. This highlights the compound effect of mental health status (SCZ vs. Healthy) and violence, and shows that the integrative role of the dACC within the emotional-salience network is particularly impaired in SCZ+V men.

Concurrently, we observed increased nodal clustering coefficients in SCZ+V men in the dACC. Given that the nodal clustering coefficient is based on the interconnectivity of a node's neighbors (59), the increased coefficient is corollary to the aberrant increased connectivity among the dACC, left rPFC, and right rPFC in schizophrenia men with a history of violence. As such, these results are consistent with previous findings of altered dorso-lateral PFC activation in violent schizophrenia subjects during the processing of angry faces (30). Within the context of emotion processing, the dorso-lateral PFC and dACC are associated with executive control processes (60). Interestingly, we found decreased connectivity between the dACC and the right putamen in SCZ-V subjects compared to Healthy participants, and an absence of connectivity in SCZ+V. A decreased/absent coupling could indicate an impairment in communication between systems implicated in cognitive control (conflict and performance monitoring) (dACC) and emotional responding in SCZ+V men. Taken together, these results might suggest compromised negative emotion regulation abilities in SCZ+V men, which is viewed as integral in the adoption of violent behavior (4). Furthermore, we observed a disrupted coupling between frontal regions and the right hippocampus specific to SCZ+V. Meta-analyses of neuro-imaging studies have shown that negative emotional stimuli elicit a decreased hippocampal activity in SCZ subjects compared to healthy controls (61). Taylor et al. (61) proposed that the hippocampus could be involved in memory retrieval during emotion processing. Therefore, altered coupling between the right hippocampus and the frontal regions could imply a difficulty in contextualizing negative emotions (62). These results suggest widespread disrupted functional connectivity within the emotional-salience network among SCZ+V men during negative emotion processing, which was posited to be an underlying mechanism in the emergence of aggressive behavior both in schizophrenia and non-psychotic populations (9, 31, 32). As we did not employ effective connectivity measures, we cannot postulate on the directionality of the impaired connection. That is, we cannot determine if results reflect an aberrant limbic drive and/or a failure of prefrontal executive control mechanisms in the context of negative stimulation (14). Nevertheless, we observed altered coupling between frontal (rPFC and dACC) and limbic (hippocampus) /striatal (putamen) regions specific to SCZ+V men. These results complement the only study to date in functional connectivity among SCZ men focusing on aggression, which revealed decreased fronto-amygdalar connectivity at rest (31), and highlight the importance of the dACC in the neurobiology of violent behavior in schizophrenia.

The ACC plays a central role in integrating negative affect and cognitive control through its ventral-affective and dorsal-cognitive subdivisions, respectively (63–65), and is therefore crucial in the neurobiology of violence and aggression (9, 32). During negative emotion processing in SCZ+V men, we have previously shown a disrupted activity of the *ventral* ACC indicating an increased neurophysiologic reactivity to emotions (29), while in the current study, we found aberrant *dorsal* ACC functional connectivity patterns consistent with an impaired cognitive control over emotions. The dACC plays a key role in processes that are critical to successful emotion regulation, namely conflict monitoring and performance (error) monitoring (66, 67), as well as emotion awareness (68). Taken together, the results from our two studies are complementary and indicate that the ACC plays a crucial role in SCZ+V men that may lead to a difficulty in integrating affective and cognitive aspects of negative emotion processing.

This study presents certain limitations. Both SCZ groups were taking antipsychotic medications which could confound the results, although no between-group differences were observed in chlorpromazine equivalent doses and the number of subjects receiving clozapine (29). This study also lacked a comparison group of non-psychotic subjects presenting a history of violence. Moreover, psychopathy was not assessed in the present study. Our analyses could have also benefited from a better characterization of the participants' violent behavior, specifically the recency of the behaviors. Furthermore, the IAPS images used in our study were not optimized to investigate discrete emotions, such as anger. Lastly, correlations were utilized as the measure of functional connectivity. Consequently, we cannot infer directionality and/or causality in the connectivity, which limits our capacity to interpret our results as we do not have an indication of brain regions inhibiting or potentiating one another.

## Conclusions

To conclude, this is the first study to characterize the alterations in functional connectivity and related topological changes of the emotional-salience network during the processing of negative emotion among men with schizophrenia with a history of violence. An increased connectivity specifically among SCZ+V men between the dACC, left rPFC, and right rPFC, and concurrently, a decrease in functional connectivity between the frontal regions (left/right rPFC and dACC) and the limbic system was observed. These alterations in connectivity translated into a higher regional clustering among the frontal regions, and a decrease in the betweenness-centrality of the dACC within the network. Together with the activation results reported previously by our research team (29), these results provide a more global picture of the brain functioning alterations during negative emotion processing in SCZ+V men, and highlight the central role of the ACC in the neurobiological bases of violent behavior in schizophrenia. Future studies using effective connectivity are needed to characterize the direction of the connectivity alteration, while paying attention to specific discrete emotions like anger.

## Data Availability Statement

The datasets for this article are not publicly available because the fMRI data were collected with formal approval from the Regroupement de Neuroimagerie du Québec, the Centre de recherche de l'Institut Universitaire en Santé Mentale de Montréal, and the Institut Philippe-Pinel de Montréal. They did not approve making anonymized data available. Requests to access the datasets should be directed to AD (alexandre.dumais@umontreal.ca).

## Ethics Statement

The studies involving human participants were reviewed and approved by Centre de recherche de l'Institut Universitaire en Santé Mentale de Montréal Regroupement de Neuroimagerie du Québec Institut Philippe-Pinel de Montréal. The patients/participants provided their written informed consent to participate in this study.

## Author Contributions

AT wrote the manuscript and did the brain imaging analyses. SP was involved in study design, brain imaging analyses, writing the manuscript, as well as provided critical comments about the manuscript. JD and CF were involved in brain imaging analyses, as well as provided critical comments about the manuscript. VZ and OL were involved in patient recruitment and assessment, as well as provided critical comments about the manuscript. AM was involved in study design, as well as provided critical comments about the manuscript. AD was involved in study design, patient recruitment and assessment, as well as provided critical comments about the manuscript. All authors contributed to and have approved the final manuscript.

### Conflict of Interest

AD and SP are co-PIs on a grant from Otsuka Pharmaceuticals and HLS Therapeutics. The remaining authors declare that the research was conducted in the absence of any commercial or financial relationships that could be construed as a potential conflict of interest.
