# Supplementary material for: Violent Behavior Is Associated With Emotion Salience Network Dysconnectivity in Schizophrenia
Source: Front Psychiatry. 2020 Feb 28;11:143. doi: 10.3389/fpsyt.2020.00143 (PMC7059347; doi:10.3389/fpsyt.2020.00143)
Supplement: Supplementary file 1 [file Data_Sheet_1.PDF]

Article Title: Violent behavior is associated with emotion salience network dysconnectivity in schizophrenia

Journal Name: Frontiers in Psychiatry

Author Names: Andràs Tikàsz, Stéphane Potvin, Jules Dugré MSc, Cherine Fahim, Vessela Zaharieva, Olivier Lipp, Adrianna Mendrek, Alexandre Dumais

Corresponding author : Alexandre Dumais, MD, PhD; Institut Philippe-Pinel de Montréal, 10905 Henri-Bourassa Est, Montréal, Québec, Canada, H1C 1H1. Tel.: (514) 648-8461. *E-mail address:* alexandre.dumais@umontreal.ca

SUPPLEMENTARY MATERIAL

Table S1. Characteristics of participants.

	SCZ+V (n=20)	SCZ-V (n=19)	Healthy controls (n=21)	Significance
Age in years, mean (SE)	30.0 (1.6)	31.4 (1.7)	30.9 (1.7)	F=0.2; P=0.842
Parental SES (SE)	3.4 (0.3)	2.9 (0.1)	2.4 (0.2)	**F=4.4; P=0.017***
Handedness, % right	90	78.9	85.7	χ^2=4.0; P=0.400
Diagnoses	6 SA	3 SA	–	χ^2=1.1; P=0.292
Age of onset in years (SE)	21.0 (1.1)	20.8 (0.8)	–	F=0.01; P=0.909
Duration of illness (SE)	9.5 (4.8)	10.6 (7.5)	–	F=0.3; P=0.609
PANSS				
Positive (SE)	9.1 (2.4)	12.1 (0.8)	–	**F=9.6; P=0.010**
Negative (SE)	12.9 (5.5)	15.5 (1.3)	–	F=2.0; P=0.166
Disorganization (SE)	6.8 (1.9)	8.5 (0.4)	–	**F=7.4; P=0.010**
Excitation (SE)	8.3 (3.0)	7.5 (0.6)	–	F=0.6; P=0.433
Depression (SE)	6.5 (2.3)	7.1 (0.4)	–	F=0.7; P=0.416
Ratings				
Positive images (SE)	4.6 (0.3)	4.8 (0.3)	4.4 (0.3)	F=0.3; P=0.784
Negative images (SE)	5.3 (0.4)	5.3 (0.3)	5.1 (0.3)	F=0.5; P=0.603
Neutral images (SE)	2.4 (0.4)	2.1 (0.5)	1.2 (0.3)	**F=2.8; P=0.072***
Chlorpromazine equivalents in mg (SE)	846.9 (170.7)	654.4 (74.7)	–	F=1.1; P=0.309
Clozapine (n)	6	9	–	χ^2=1.2; P=0.265

Significant results are shown in bold (P<0.05). *SCZ−V > Controls (P<0.05).

Abbreviations: PANSS, Positive and Negative Syndrome Scale; SA, schizoaffective disorder; SCZ+V, schizophrenia with violent behavior; SCZ−V, schizophrenia without violent behavior; SE, standard error; SES, socioeconomic status (a higher number indicates a lower SES).

Table taken from:

Tikasz, A., Potvin, S., Lungu, O., Joyal, C. C., Hodgins, S., Mendrek, A., et al. (2016). Anterior cingulate hyperactivations during negative emotion processing among men with schizophrenia and a history of violent behavior. Neuropsychiatr Dis Treat, 12, 1397-1410, doi:10.2147/NDT.S107545.

Table S2. Group mean Fisher's Z-score (Standard Error of the Mean) for ROI-to-ROI functional connectivity during negative emotion processing.

Seed ROI	Target ROI	Healthy		SCZ-V		SCZ+V	
Putamen r							
	dACC	0.376	(0.178)	0.254	(0.241)	0.055	(0.213)
	rPFC l	0.186	(0.187)	0.193	(0.194)	0.029	(0.219)
dACC							
	Putamen r	0.376	(0.178)	0.254	(0.241)	0.055	(0.213)
	Putamen l	0.353	(0.191)	0.230	(0.237)	0.078	(0.226)
	Hippocampus r	0.044	(0.145)	0.045	(0.341)	-0.184	(0.225)
	rPFC l	0.730	(0.194)	0.775	(0.256)	0.957	(0.307)
rPFC l							
	Hippocampus r	-0.082	(0.175)	-0.049	(0.264)	-0.266	(0.177)
	rPFC r	0.820	(0.245)	0.877	(0.295)	1.123	(0.325)
	Hippocampus l	-0.097	(0.178)	-0.015	(0.235)	-0.250	(0.210)
	dACC	0.730	(0.194)	0.775	(0.256)	0.957	(0.307)
	Putamen r	0.186	(0.187)	0.193	(0.194)	0.029	(0.219)
Putamen l							
	dACC	0.353	(0.191)	0.230	(0.237)	0.078	(0.226)
rPFC r							
	rPFC l	0.820	(0.245)	0.877	(0.295)	1.123	(0.325)
	Hippocampus r	-0.070	(0.128)	-0.071	(0.258)	-0.254	(0.213)
Hippocampus l							
	rRPFC l	-0.097	(0.178)	-0.015	(0.235)	-0.250	(0.210)
Hippocampus r							
	rPFC l	-0.082	(0.175)	-0.049	(0.264)	-0.266	(0.177)
	dACC	0.044	(0.145)	0.045	(0.341)	-0.184	(0.225)
	rPFC r	-0.070	(0.128)	-0.071	(0.258)	-0.254	(0.213)

Abbreviations: ACC, Anterior cingulate cortex; PFC, prefrontal cortex; ROI, region of interest; r, right; l, left.
